# The Effect of Shelter on Oxidative Stress and Aggressive Behavior in Crested Newt Larvae (*Triturus* spp.)

**DOI:** 10.3390/ani10040603

**Published:** 2020-04-01

**Authors:** Tamara G. Petrović, Tijana Z. Vučić, Sonja Z. Nikolić, Jelena P. Gavrić, Svetlana G. Despotović, Branka R. Gavrilović, Tijana B. Radovanović, Caterina Faggio, Marko D. Prokić

**Affiliations:** 1 Department of Physiology, Institute for Biological Research “Siniša Stanković”, National Institute of Republic of Serbia, University of Belgrade, 11060 Belgrade, Serbia; tamara.petrovic@ibiss.bg.ac.rs (T.G.P.); jelena.gavric@ibiss.bg.ac.rs (J.P.G.); despot@ibiss.bg.ac.rs (S.G.D.); perendija@ibiss.bg.ac.rs (B.R.G.); tijana@ibiss.bg.ac.rs (T.B.R.); marko.prokic@ibiss.bg.ac.rs (M.D.P.); 2 Faculty of Biology, Institute for Zoology, University of Belgrade, 11000 Belgrade, Serbia; tijana.vucic@bio.bg.ac.rs (T.Z.V.); sonjadj@bio.bg.ac.rs (S.Z.N.); 3 Department of Evolutionary Biology, Institute for Biological Research “Siniša Stanković”, National Institute of Republic of Serbia, University of Belgrade, 11060 Belgrade, Serbia; 4 Department of Chemical, Biological, Pharmaceutical and Environmental Sciences, University of Messina, 98122 Messina, Italy

**Keywords:** amphibian larvae, antioxidant system, oxidative stress, hybrid, refuge

## Abstract

**Simple Summary:**

A lack of adequate refuge, even in the absence of predators, can alter the metabolism, levels of corticosteroids, growth and behavior in various groups of animals. Even though some of those processes (higher metabolic rates and activation of the stress response of the hypothalamic-pituitary-adrenal axis) in animals can result in enhanced reactive species (RS) production and oxidative stress, there is no study examining the effects of shelter on oxidative stress parameters. The results from this study showed that in crested newt larvae the absence of refuges altered their oxidative/antioxidative status and movement, but did not affect their aggressivity/injuries rate. Higher values of catalase, glutathione peroxidase, glutathione S-transferase and glutathione can indicate increased production of hydrogen peroxide in individuals without an adequate hiding place. This boosted antioxidant defense has a certain physiological cost that can be expressed in terms of the consumption of energy needed to maintain it as upregulated.

**Abstract:**

Shelters are important for animal survival. Provision of adequate hiding places allow animals to express their natural sheltering behavior and it can have different positive effects on cortisol levels, physiological processes and mental performance. Although the absence of a refuge activates some stress response, its effect on oxidative stress has not been adequately examined. This study investigated whether the presence/absence of a shelter modifies the oxidative status (the antioxidant system and oxidative damage) and aggressive behavior of crested newt larvae (*Triturus macedonicus* and its hybrid with *T. ivanbureschi*). Our results show that individuals reared with shelters had lower values of the tested antioxidant parameters (catalase, glutathione peroxidase, glutathione S-transferase and glutathione), indicating a lower production of reactive species than individuals reared without shelter. The same pattern was observed in both *T. macedonicus* and its hybrid. Contrary to the activation of some physiological pathways, shelter availability did not significantly affect the rate of intraspecific aggressive behavior. The physiological benefits of shelter use can be manifested as a lower requirement for investment in the energy necessary for the maintenance of the upregulated antioxidant defenses, activation of repair systems and synthesis of endogenous antioxidants. This study highlights the importance of shelter provision, which may be valuable in habitat restoration and animal conservation studies.

## 1. Introduction

The existence of shelters has been shown to be of great importance for the survival of animals that remain hidden for significant amounts of time during different processes (e.g., rest, sleep, digestion, reproduction) [1,2,3]. Shelters provide several benefits, such as protection from other animals to avoiding adverse environmental conditions [4,5,6]. For example, shelter nest-box use in birds reduce their thermoregulatory costs, allowing for the allocation of stored resources to egg production [7]. For animals that need to hide from predators, shelters provide safety and enable the saving of energy necessary for camouflage or vigilance. Abandonment of shelters or a lack thereof exposes individuals to challenging and sometimes hostile abiotic and biotic factors [4,5,8]. Under the latter scenario, individuals are forced to move to avoid adverse environmental factors, which can affect other animal activities. Exposed individuals are in constant preparation for explosive and/or prolonged physical activities (e.g., swimming, running), increased mental alertness and maintenance of all senses at a level for a fast reaction [9,10]. All of these activities are metabolically demanding and can alter the energy budget, behavior and physiological processes [2,6,11,12]. It was shown that the lack of shelter elevated the basal corticosterone level in snakes [2], while in the Atlantic salmon *Salmo salar* no access to shelter increased their basal plasma cortisol levels, standard metabolic rates and overall metabolism [6,11].

Even though higher metabolic rates and activation of the stress response of the hypothalamic–pituitary–adrenal (HPA) axis [13,14] in animals devoid of adequate hiding places result in enhanced reactive species (RS) production and oxidative stress, to our knowledge there is no study examining the effects of shelter on oxidative stress parameters. Managing RS production is a pivotal physiological process, as unquenched RS can wreak havoc on cell components (lipids, proteins and DNA) and structures, and negatively affect individual fitness [15]. The antioxidant defense (AOS) machinery plays an important role in this process. The system consists of a complex of enzymatic and non-enzymatic components, which together are involved in the lowering and removal of RS and/or transforming them into less reactive compounds. They also eliminate intermediate derivatives of oxidative damage (hydroperoxides) from the organism [16].

Amphibian populations are in decline worldwide and more than 40% of all known amphibian species are marked as threatened by extinction [17]. Several factors (habitat modification, environmental pollution, climate change, invasive species and pathogens) and their synergic effects have been suggested as responsible for this situation [17]. Habitat modification is among the most important causes of many declines, highlighting the importance of shelter presence [18]. Even though all life stages are affected by these factors, the development of larvae and their metamorphosis can be additionally sensitive to unpredictable and variable conditions that can significantly affect individual survival and fitness (body condition, length of the development period, morphology and behavior) [19,20,21,22,23]. The putative carry-over effects of having a poorer performance/physiological status at the early stages are not well understood yet, and compensatory responses might leave negative consequences on the life history of an individual in the long run.

Other than habitat deterioration, amphibian larvae are also vulnerable to predation by fish and aquatic invertebrates. For newts, the presence of fish was shown to be particularly detrimental. Several studies have demonstrated that appropriate hiding places can mitigate these threats. It was suggested that supporting the aquatic vegetation should be favored as one of the effective conservation measures for newts [24]. Refuge availability has been found to increase the survival rate of two newt species’ (*Lissotriton helveticus* and *Ichthyosaura alpestris*) larvae by more than twofold when the predator (brown trout *Salmo trutta*) was present [25]. A complex habitat reduces the predator–prey encounter rate, important in antipredator behavior in the presence of visually oriented predators such as fish [26]. The positive effect of shelter provision on amphibian larvae fitness was also reported for the red-eyed treefrog (*Agalychnis callidryas*). Larvae kept in a shelter showed signs of reduced stress and metamorphosed having significantly larger body sizes and did so at a later time than individuals reared either without shelter or only with shade [12].

Alongside the threat of predation, intraspecific aggression represents another major source of mortality for newt larvae [27]. Conspecific attacks often result in injuries of the tail, gills or limbs [28,29], and cannibalism is also common [28]. This aggressive behavior lowers intraspecific competition as the competitive ability or survival of injured larvae is reduced when compared to that of uninjured organisms [29]. It was suggested that intraspecific aggression tends to decrease when larvae face stressful environments/conditions, such as drying environments and the presence of predators [28,30]. The challenges that newt larvae face during development (predation, interspecific aggression and habitat modification) make them a suitable model organism for studying the effects of environmental changes. Their ability to hybridize provides an additional possibility to examine potential differences between parental species and hybrids in response to those changes. In general, data on the biochemical and physiological parameters of newts are scarce. A better understanding of these parameters could facilitate an explanation of species interactions in natural populations.

The main objective of the present study was to investigate the possible effects of the presence/absence of shelter on the oxidative status (antioxidant defense system and oxidative damage) of crested newt larvae (*Triturus macedonicus* and its hybrid with *T. ivanbureschi*). We also wanted to investigate the possible effects of shelter on the behavior (movement and aggression) of the larvae. Our overall expectation was that the presence of a shelter would benefit newt larvae and that the sense of safety would positively affect their oxidative status (lower oxidative stress) on the one hand, while the more shelter-devoid larvae would display higher levels of intraspecific aggression (a greater number of injuries) on the other, as a result of competition for shelter.

## 2. Materials and Methods

### 2.1. Experimental Design

Newts of the genus *Triturus* belong to a monophyletic group of nine species widely distributed across western Eurasia, which occupy a range of different habitats and make several hybrid zones [31,32,33,34]. Larvae used in this study were obtained from breeding *T. macedonicus* and *T. ivanbureschi* parental individuals from natural populations outside the hybrid zone with known genetics [31,32,33,34]. The breeding took place in March 2018 after a period of hibernation in a cold chamber at a constant temperature (4 °C). The larvae, having reached developmental stage 62, were randomly chosen and sequestered into one of two experimental groups: (i) no shelter or (ii) with shelter. Twelve 10 L aquariums (30 × 20 × 20 cm) were used, six with shelters and six without. We placed 12 individuals in each aquarium—144 individuals in total. Larvae density was chosen according to the natural densities range of closely related newt species [35] in order to obtain the optimal frequency of individual interactions. Developmental stage 62 was recognized according to the formation of the fifth digit on the hindlimb, which marks a fully formed extremity and complete tail development [36]. From stage 62 onwards, larvae only increase in size until the end of metamorphosis. Differences in larval tail shape and size between parental species and hybrids raised in experimental conditions were observed at this stage [37].

For shelter provision, we used PVC tubes of 12.5 mm in diameter. The tubes were cut in 10 cm-long sections (total length of 1.5 m) and were placed in shelter aquariums. The tubes were bundled together, forming structures of 3 × 3 and 3 × 2 tubes. The structures covered about one-third of the aquarium floor area, leaving enough open and hiding space for the individuals (Supplementary Figure S1).

The larvae were reared in aerated dechlorinated tap water under an ambient photoperiod. The water temperature was kept stable (18–19 °C). Every second day, the larvae were fed with *Tubifex* sp. The water in the aquariums was changed and the tubes were cleaned two or three times a week. The experiment lasted 30 days. During the experiment we followed shelter use and movement activity, while the injuries/aggressive behavior and oxidative status of the individuals were obtained after.

To determine the snout-vent length (SVL) and to visualize the number of possible injuries (Figure S2), all larvae were photographed at the end of the experiment using a Sony DSC-F828 digital camera (24-bit color and 3264 × 2448 pixel resolution, MP; Sony Corp., Tokyo, Japan). Larval length was calculated as the distance from the snout tip to the outer edge of the cloaca using the TMorphGen6 program from the IMP package [38]. In order to check if larvae used the provided shelters, we recorded the number of individuals in shelters twice every day at the same time. A larva was marked as in shelter if its entire body was inside the tube.

To test the activity of the larvae, we placed randomly chosen individuals in a plastic tank (size 18 cm × 10 cm) and calculated the number of lines crossed, defined by the entire body and tail crossings of the line as in Crane et al. [39]. Below the tank, a paper with a drawn network of 9 cm × 5 cm squares was laid. A larva was left to acclimate for 5 min and during the next 2 min the numbers of crosses were quantified. This test was performed twice and in duplicate, always at the same time of day, in the evening between 8 and 10 p.m. when the newts were usually active. In this test, we included six randomly chosen individuals from each of the 12 aquariums (72 larvae in total). The activity test was performed in the last week of the experiment, in order to obtain the maximal effects of shelter absence/provision on the studied performance.

At the end of the experiment, the larvae were killed by placing them in liquid nitrogen and were kept at −80 °C until further analyses [40,41].

The collecting of animals for the experiment was approved by the Ministry of Energy, Development and Environmental Protection of the Republic of Serbia (permit no. 353-01-75/2014-08) and the Environmental Protection Agency of Montenegro (permit no. UPI-328/4). The experimental procedure was approved by the Animal Ethical Committee of the Institute for Biological Research “Siniša Stanković”, University of Belgrade (decision no. 03-03/16).

### 2.2. Sample Processing

Whole bodies of larvae were first finely chopped and mixed to obtain material that was as homogenous as possible. After that, a part was taken for the determination of thiobarbituric acid-reactive substance (TBARS) concentration (lipid peroxidation (LPO)), while the rest was used for the antioxidant parameters and protein carbonylation (PC) determination [42]. The larvae were homogenized (Ultra Turrax homogenizer T-18, IKA-Werk, Germany) in a 1:5 ratio in an ice-cold 25 mM sucrose buffer (pH 7.4) containing 10 mM Tris-HCl and 5 mM EDTA to disrupt the cell membranes and to release the cytosolic fraction [42]. Thereafter, the homogenates were sonicated with an ultrasonic homogenizer (Sonopuls HD 2070, Bandelin electronic, Germany) for 30 s at 10 kHz on ice to break the subcellular structures. One part of each sonicate was centrifuged at 5000 × *g* for 10 min in 10% sulfosalicylic acid and the resulting supernatants were used for determination of GSH, while the rest was placed in tubes and centrifuged in a L7-55 ultracentrifuge (Beckman, USA) at 100,000 × *g* at 4 °C for 90 min [43]. The supernatants obtained after the ultracentrifugation process were used for measuring all other AOS parameters.

### 2.3. Biochemical Analyses

The protein concentrations in the samples were recorded using the method described by Lowry et al. [44]. Superoxide dismutase (SOD) activity was determined by autoxidation of adrenaline to adrenochrome, as described by Misra and Fridovich [45]. The rate of hydrogen peroxide (H_2_O_2_) decomposition was used to measure catalase (CAT) activity [46]. The method by Tamura et al. [47] was applied for glutathione peroxidase (GSH-Px) activity, by tracking the reduction of t-butyl hydroperoxide with nicotinamide adenine dinucleotide phosphate (NADPH). Measurement of glutathione reductase (GR) activity was based on the reduction of glutathione disulfide (GSSG) to reduced GSH using NADPH as a substrate [48]. The reaction of the -SH group of GSH with 1-chloro-2,4-dinitrobenzene (CDNB) was used for determination of glutathione S-transferase (GST) activity [49]. The activities of all enzymes were expressed as U/mg protein.

For measuring GSH, we used the method described by Griffith [50]. The concentration of GSH was determined after oxidation of GSH using 5,5´-dithiobis-(2-nitrobenzoic acid) (DTNB) and reduction by NADPH in the presence of GR. The concentrations of the total -SH groups were assayed according to Ellman’s method [51]. For measurement of the non-protein -SH groups, proteins were precipitated by sulfosalicylic acid. Protein SH groups were calculated as the differences between the total and non-protein -SH group concentrations. The level of LPO and the carbonyl content of the proteins served as markers of oxidative damages. The concentration of TBARS was measured after treating the samples with cold thiobarbituric acid reagent (10% trichloroacetic acid, 0.6% thiobarbituric acid) and heating at 100 °C [52]. The level of protein carbonylation (PC) was determined according to the 2,4-dinitrophenylhydrazine (DNPH) alkaline method [53]. More information about the biochemical analyses are given in the Supplementary Material.

All measurements were performed at 19 °C with a Shimadzu UV 1800 UV–VIS spectrophotometer with a temperature-controlled cuvette holder. Wavelengths for biochemical analyses were as follows: for SOD—480 nm; for CAT—240 nm; for GSH-Px, GR and GST—340 nm; for GSH and SH groups—412 nm; for TBARS/LPO—532 nm; and for PC—450 nm.

### 2.4. Statistical Analyses

Possible outliers, the distribution of the data and the homogeneity of variance were checked by Grubb, Kolmogorov–Smirnov and Levine tests, respectively. All data for the oxidative stress parameters met the assumption of homogeneity of variance and had a normal distribution. Generalized linear models (GLMs) were used to assess the relationships between each oxidative parameter and the variables “species” (*T. macedonicus* and its hybrid) and treatment (shelter and no shelter), as well as their interaction (the term “species” was used for easier presentation of the results).

A normal error function and an identity-link function were applied. The preliminary analyses did not reveal any significant differences between aquariums for each treatment for each oxidative stress parameter analyzed (*p* ≥ 0.11, Supplementary Table S1); thus, this factor was not retained in the analyses in order to minimize the number of variables that were included in the model [54]. Outcomes with the aquarium as a factor were similar to the given one.

The post-hoc Tukey’s HSD test was performed to test the effects of the treatments (shelter and no shelter) for *T. macedonicus* and the hybrid. Nonparametric tests were applied for analyses of shelter use and the number of injuries (Fisher exact test), as well as for activity (Mann-Whitney U test). In order to determine the possible relationship/correlations between the individual’s SVL and the number of injuries, Spearman rank correlations were applied. In all tests, *p* < 0.05 was chosen as the criterion for statistical significance. To investigate the variation in AOS parameters within and between the examined groups, we used canonical discriminant analysis (CDA). All analyses were done in STATISTICA 8.0 [55].

## 3. Results

### 3.1. Oxidative Stress

Average snout-vent lengths (SVL) ± standard deviations and the SVL minimum and maximum values for the individuals under each treatment were as follows: *T. macedonicus*/no shelter—2.45 ± 0.19 cm (SVL min. 2.10 and max. 2.86); *T. macedonicus*/shelter—2.46 ± 0.22 cm (1.95 and 2.84); hybrid/no shelter—2.64 ± 0.15 cm (2.16 and 2.91); and hybrid/shelter—2.70 ± 0.19 cm (2.01 and 2.97).

The GLMs revealed significant differences between *T. macedonicus* and the hybrid, between individuals reared in shelter vs. no-shelter conditions, as well as their interaction (Table 1). The parameters that differed both between species and between treatments were CAT, GSH-Px, GST and GSH. The between-species comparison also showed significant differences in SVL, -SH group concentrations and GR activity, while between treatments LPO concentrations differed. A significant interaction, species × treatments, was only reported for the activity of GR.

**Figure 1:**
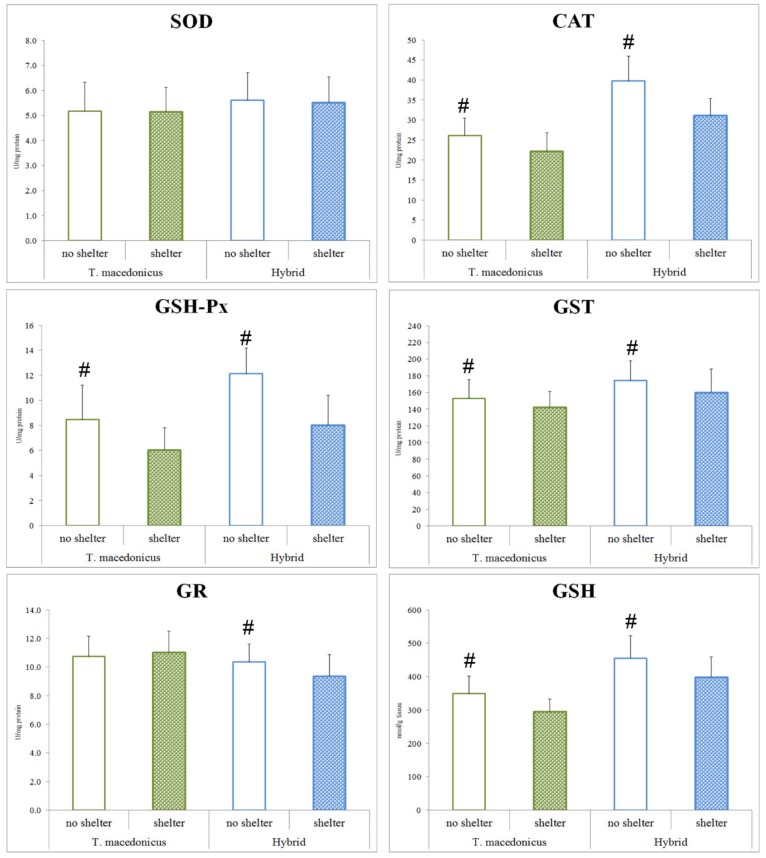
Antioxidant system parameters (SOD, CAT, GSH-Px, GST, GR and GSH) in treatments (shelter and no-shelter conditions) for *T. macedonicus* and hybrid individuals. All data are presented as the mean ± standard error. Significant differences (*p* < 0.05) between individuals from the shelter and no-shelter experiments are marked with “#”. SOD—superoxide dismutase; CAT—catalase; GSH-Px—glutathione peroxidase; GST—glutathione S-transferase; GR—glutathione reductase; GSH—glutathione.

**Figure 2:**
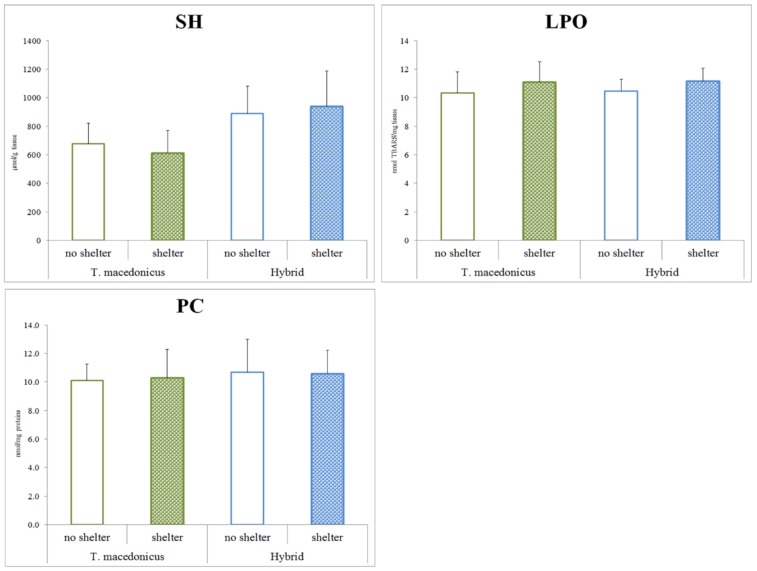
The protein -SH group concentrations and oxidative damage parameters (TBARS and PC) in treatments (shelter vs. no shelter) for *T. macedonicus* and hybrid individuals. All data are presented as the mean ± standard error. Significant differences (*p* < 0.05) between individuals from the shelter and no-shelter conditions are marked with “#”. SH—sulfhydryl groups; LPO—lipid peroxide; PC—protein carbonylation.

As the main aim of this study was to examine the possible effects of shelter on the chosen oxidative stress parameters, we focused on the differences between the treatments for each species separately (*T. macedonicus* and its hybrid: Figure 1 and Figure 2). In *T. macedonicus* individuals with shelter, we found significantly lower activities of CAT (*p* < 0.001), GSH-Px (*p* < 0.001) and GST (*p* = 0.044), and a lower concentration of GSH (*p* < 0.001) in comparison to those from no-shelter conditions. For hybrid individuals, the presence of shelter led to lower activities of CAT (*p* < 0.001), GSH-Px (*p* < 0.001), GST (*p* = 0.011) and GR (*p* = 0.004), and to a lower concentration of GSH (*p* = 0.005). We did not observe significant differences between individuals reared in no-shelter and shelter conditions for oxidative damage parameters, e.g., concentrations of LPO (*T. macedonicus p* = 0.159; hybrid *p* = 0.081) and protein carbonyls (*T. macedonicus p* = 0.661; hybrid *p* = 0.951). No differences were also found for SOD activity (*T. macedonicus p* = 0.928; hybrid *p* = 0.771) and -SH group (*T. macedonicus p* = 0.092; hybrid *p* = 0.349) concentrations in the examined species.

**Figure 3:**
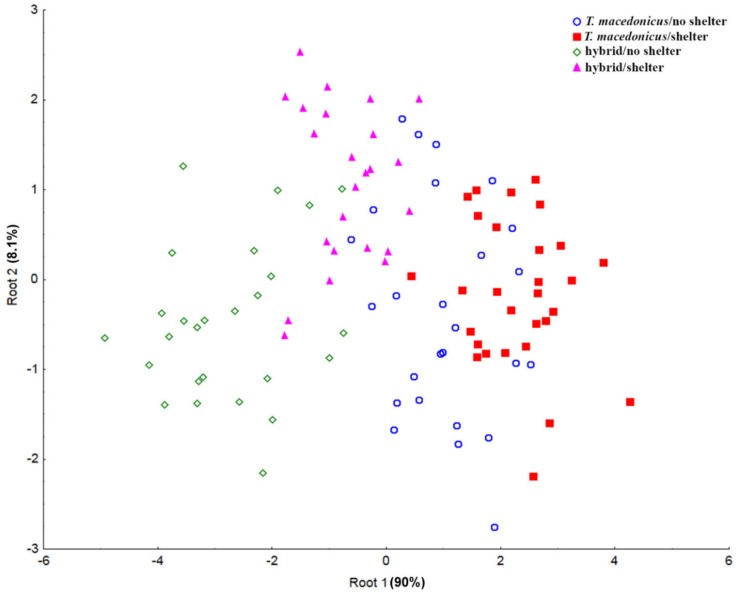
Canonical discriminant analyses of the antioxidant parameters (SOD, CAT, GSH-Px, GST, GR and GSH) for the examined groups. SOD—superoxide dismutase; CAT—catalase; GSH-Px—glutathione peroxidase; GST—glutathione S-transferase; GR—glutathione reductase; GSH—glutathione.

Based on CDA, we observed a clear separation only along Root 1. The separation emerged between the examined species (*T. macedonicus* vs. hybrid individuals), and the parameters that contributed most to this separation were CAT, GSH and GR (Figure 3 and Supplementary Material Table S2).

### 3.2. Shelter Use, Movement and Aggressive Behavior

Data about the numbers of individuals using shelter revealed that during the experiment, on average 44.1% of the *T. macedonicus* individuals and 30.8% of the hybrid individuals were hidden. Most of the remaining individuals of both species remained near the shelters. Comparison between species showed that *T. macedonicus* individuals tended to use shelter more often than the hybrids (*p* = 0.035).

**Figure 4:**
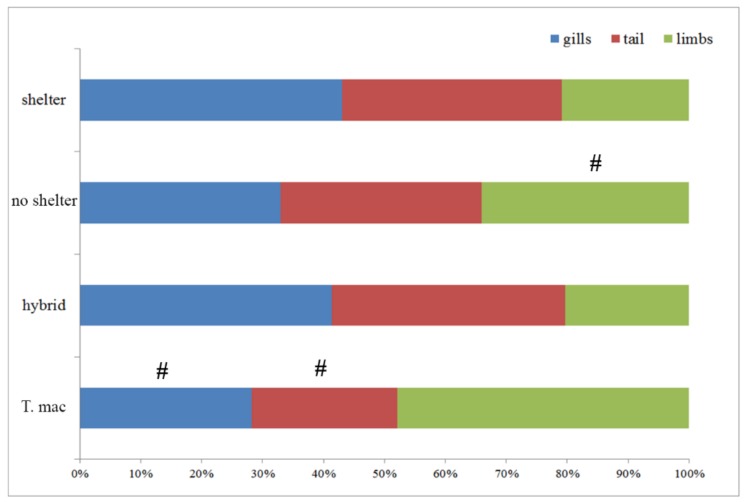
The number of body regions injured (gills, tail and limbs) in different treatments (shelter vs. no shelter) and between *T. macedonicus* and hybrid individuals given as percentages. A # marks statistical difference.

The percentages, i.e., ratios of individuals with injuries in different experimental groups, were as follow: sheltered *T. macedonicus*, 41.6%; without shelter *T. macedonicus*, 55.5%; sheltered hybrids, 94.4%; and without shelter hybrids, 86.1%. Within the species, the differences in numbers of injured individuals between treatments were not significant (for *T. macedonicus p* = 0.181; hybrid *p* = 0.213). Comparison of the numbers of individuals with injuries between the species for each treatment showed significant differences (for shelter *p* < 0.000 and for no shelter *p* = 0.004). The hybrids contained a greater number of injured individuals. When the number of injuries was correlated with body length for all the examined individuals a significant correlation was observed (r = 0.26, *p* = 0.01); however, further analyses did not show significant correlations with the body length and injuries of the larvae within treatments (*T. macedonicus* shelter r = −0.04, *p* = 0.846; *T. macedonicus* without shelter r = −0.29, *p* = 0.169; hybrid shelter r = −0.340, *p* = 0.104; hybrid without shelter r = −0.328, *p* = 0.126). We also examined which parts of the body were injured in each group, e.g., tail, limbs and gills. The results are presented in Figure 4 and Table S3.

The percentages of individuals that crossed lines during the activity test were as follows: for sheltered *T. macedonicus*—61.1%; no shelter *T. macedonicus*—69.4%; sheltered hybrids—80.5%; no-shelter hybrids—100%. The differences in the numbers of crosses between treatments were significant only for the hybrid species. A significant difference could be observed also between the species for each treatment (Table 2).

## 4. Discussion

Newt larvae primarily use shelters to thermoregulate and to avoid predators [24]. In general, they spend a significant amount of time resting in or near shelters. In the presence of predators, this time increases to the point that it can affect normal feeding behavior and growth tempo [30]. In the present study between one-third and one-half of all the individuals were hiding in shelters during the check, which is in concordance with results from other studies [56,57]. This finding confirmed that shelter availability can be marked as one of the favorable factors for normal newt development [25].

Evidence has been presented showing that a lack of adequate refuge, even in the absence of predators, can alter metabolism, the levels of corticosteroids, growth, behavior and, as we assumed, the oxidative status in various groups of animals (fish, snakes, birds and amphibians) [11,12,26]. Alternations in oxidative stress reported in this study were observed through changes in the antioxidant system rather than through direct oxidative damage. Newts that developed in no-shelter conditions had higher values of AOS components (GSH, CAT, GSH-Px and GST). The same pattern was confirmed for both the *T. macedonicus* and hybrid individuals. The boosted first line of the AOS (CAT and GSH-Px) was involved in the suppression or prevention of RS formation, and the second line of defense (GSH), which removes radicals, inhibits the initiation and propagation reactions, implying that individuals reared without shelters can exhibit increased production of RS [16]. Based on the main function of CAT and GSH-Px, we can mark hydrogen peroxide as the RS that contributed most to the obtained results. In the process of removal of H_2_O_2_, GSH can be also included as a cofactor for GSH-Px activity, while GST plays a role in the defense against oxidative damage and peroxidation of DNA and lipids [58]. In the case of hybrid individuals, the GSH system was enhanced, including increased activity of GR, which maintains higher levels of GSH. A higher RS concentration in no-shelter-reared newts can be linked with higher corticosterone levels and metabolic rates caused by shelter absence.

The relationship between shelter and metabolism has already been documented for some fish species [6,59,60]. Fish without shelter had an average 30% increase in metabolic costs and on average two to three times higher basal levels of plasma cortisol than fish from the enrichment treatments [11]. The shelter also had a major influence on the cortisol response to stress in silver catfish (*Rhamdia quelen*) [61]. Corticosterone increase was also observed in snakes without shelter [2]. Provision of the opportunity to shelter as a naturally preferred behavior can lead to physiological relaxation or a calming effect. Distress is often associated with a lower predation risk and decreased vigilance (a heightened state of body awareness in response to predation), which may in turn elevate opercular ventilation and ultimately metabolism [6].

The higher activity of AOS in order to sustain RS seen in individuals without shelters can also have its costs. The physiological cost can be expressed in terms of the consumption of energy needed to maintain the upregulated antioxidant defenses, to activate the repair systems, to synthesize endogenous antioxidants (such as GSH) as well as to increase the dietary intake of exogenous antioxidants [15,62]. The required energy can be diverted from other processes and can further affect the animal’s fitness [63,64,65,66]. This can be more pronounced in natural conditions under which the animals are potentially limited by the available feeding time, food availability, capacity to process energy, predation and other abiotic and biotic factors.

Our second expectation was that shelters would affect the behavior (aggression and movement) of crested newt larvae. Previous studies showed that conspecific aggressive behavior of newt larvae can decrease significantly when they are challenged with unfavorable conditions [28,30]. Larvae tend to lower intraspecific aggression under such circumstances, and this led us to the assumption that physiologically relaxed shelter individuals would display higher levels of aggressive behavior, e.g., a higher number of injuries, which can be accompanied by increased competition for shelter use. Shelter presence or absence in this study did not significantly affect the number of injuries, indicating that shelter as such does not modify the intraspecific competition of newt larvae. On the other hand, the activity test revealed that individuals reared without shelter tended to be more active when placed in a new environment in comparison to those reared with shelter. Lower movement levels can be the result of sheltering behavior developed in individuals that were provided with shelter. Lower movement in some amphibian larvae is also considered as an antipredator response [39]. The comparison of *T. macedonicus* and its hybrid revealed that hybrid individuals were significantly more aggressive and active in both treatments. They also tended to use less shelter. Thus, we found indications for more intense competition among hybrid individuals. The reasons for these differences could be found in differences in metabolic rates. Our previous study on the same hybrid suggested that hybrid individuals can have a higher metabolic rate compared to their parental species due to mitonuclear mismatch [66]. Higher metabolic rates in hybrid newts were also reported by Gvoždík [67]. According to the “increased intake hypothesis”, individuals with higher standard metabolic rates have greater needs for energy, are exposed to greater competition (dominance and aggressiveness), attain larger body sizes, but also exhibit higher foraging rates and activity levels [68]. In this study, hybrid individuals had significantly higher values of SVL in comparison to *T. macedonicus* individuals. Hybrid individuals also displayed more injuries, mostly in the gills and tail region. The reason for the higher number of tail injuries can be due to the tail filament that hybrid individuals have in comparison to *T. macedonicus* [37], which may lure other individuals to attack it more frequently [69]. Injured larvae can be more susceptible to infections, cannibalism or predation as a result of the compromised function of the injured body parts (locomotion and respiration) and the energy invested in their regeneration [30].

## 5. Conclusions

We believe that this investigation is an important addition to experimental studies that have been carried out on different groups of organisms (fish, snakes and amphibians) that point to the negative effects of shelter absence and its association with increased stress levels. The absence of refuges for crested newt larvae altered their oxidative/antioxidative status and movement but was not related to their aggressivity/injury rates. All these results indicate that shelter should be included as one of the crucial factors in habitat assessment and restoration, animal welfare and field management, in both captivity experiments and conservation studies.

The questions that remain to be clarified in future studies are how the shelter absence or presence affect the oxidative stress of larvae in harsher and more dynamic natural conditions; the possible long-term or delayed adverse effects of increased investment in the AOS on newts fitness; and the effects on the whole hybrid complex.

## Figures and Tables

**Table 1:** General linear models used to detect the significant differences in body size (snout-vent length—SVL) and oxidative status parameters between “species” (*T. macedonicus* and its hybrid) and treatments (shelter vs. no shelter). Significant *p*-values (*p* < 0.05) are given in boldface. SOD—superoxide dismutase; CAT—catalase; GSH-Px—glutathione peroxidase; GST—glutathione S-transferase; GR—glutathione reductase; GSH—glutathione; SH—sulfhydryl groups; LPO—lipid peroxides; PC—protein carbonylation.

Variable	Factor	Wald	*p*
SVL	**Species**	**39.12**	**<0.001**
	Treatments	1.40	0.236
	Species × treatments	0.92	0.338
SOD	**Species**	**4.97**	**0.025**
	Treatments	0.07	0.781
	Species × treatments	0.02	0.886
CAT	**Species**	**157.44**	**<0.001**
	**Treatments**	**44.23**	**<0.001**
	Species × treatments	1.76	0.184
GSH-Px	**Species**	**46.23**	**<0.001**
	**Treatments**	**59.78**	**<0.001**
	Species × treatments	0.54	0.461
GST	**Species**	**22.30**	**<0.001**
	**Treatments**	**10.96**	**0.001**
	Species × treatments	0.30	0.576
GR	**Species**	**19.12**	**<0.001**
	Treatments	2.69	0.101
	**Species × treatments**	**7.55**	**0.006**
GSH	**Species**	**113.72**	**<0.001**
	**Treatments**	**35.40**	**<0.001**
	Species × treatments	0.31	0.575
SH	**Species**	**61.26**	**<0.001**
	Treatments	0.26	0.609
	Species × treatments	3.15	0.075
LPO	Species	0.11	0.743
	**Treatments**	**5.25**	**0.022**
	Species × treatments	0.01	0.926
PC	Species	2.23	0.135
	Treatments	0.06	0.800
	Species × treatments	0.12	0.730

**Table 2:** Median number of lines crossed during the activity test. Significant *p*-values (*p* < 0.05) are given in boldface.

	Group	Median	*p*
***T. macedonicus***	Shelter	1.73	0.578
	No shelter	2.75	
**Hybrid**	**Shelter**	**5.00**	**<0.001**
	**No shelter**	**12.25**	
**Shelter**	***T. macedonicus***	**1.73**	**0.048**
	**Hybrid**	**5.00**	
**No shelter**	***T. macedonicus***	**2.75**	**<0.001**
	**Hybrid**	**12.25**	

## Supplementary Materials

The following are available online at https://www.mdpi.com/2076-2615/10/4/603/s1, Biochemical analyses, Table S1: *p*-values of between aquarium comparisons for each treatment for body length and oxidative stress parameters (one way ANOVA)., Table S2: Standardized canonical discriminant function coefficient for the antioxidative parameters., Table S3: Number of individuals with injuries of different body parts (gills, tail, limbs)., Figure S1: Aquariums with shelter (left) and no shelter (right) conditions., Figure S2: Injuries of gills (A), limbs (B) and tail (C).
