# Supplementary material for: The Effect of Shelter on Oxidative Stress and Aggressive Behavior in Crested Newt Larvae (Triturus spp.)
Source: Animals (Basel). 2020 Apr 1;10(4):603. doi: 10.3390/ani10040603 (PMC7222759; doi:10.3390/ani10040603)
Supplement: Supplementary file 1 [file animals-10-00603-s001.pdf]

The Effect of Shelter on Oxidative Stress and Aggressive Behavior in Crested Newt Larvae (*Triturus* spp.)

Tamara G. Petrović [1,†], Tijana Z. Vučić [2,3], Sonja Z. Nikolić [2], Jelena P. Gavrić [1], Svetlana G. Despotović [1], Branka R. Gavrilović [1], Tijana B. Radovanović [1], Caterina Faggio [4,*] and Marko D. Prokić [1,†]

[1] Department of Physiology, Institute for Biological Research "Siniša Stanković", National Institute of Republic of Serbia, University of Belgrade, 11060 Belgrade, Serbia; tamara.petrovic@ibiss.bg.ac.rs (T.G.P.); jelena.gavric@ibiss.bg.ac.rs (J.P.G.); despot@ibiss.bg.ac.rs (S.G.D.); perendija@ibiss.bg.ac.rs (B.R.G.); tijana@ibiss.bg.ac.rs (T.B.R.); marko.prokic@ibiss.bg.ac.rs (M.D.P.)

[2] Faculty of Biology, Institute for Zoology, University of Belgrade, 11000 Belgrade, Serbia; tijana.vucic@bio.bg.ac.rs (T.Z.V.); sonjadj@bio.bg.ac.rs (S.Z.N.)

[3] Department of Evolutionary Biology, Institute for Biological Research "Siniša Stanković", National Institute of Republic of Serbia, University of Belgrade, 11060 Belgrade, Serbia

[4] Department of Chemical, Biological, Pharmaceutical and Environmental Sciences, University of Messina, Messina, Italy

* Correspondence: cfaggio@unime.it.

† Authors with equal contribution

Supplementary:

Biochemical analyses

The concentration of total proteins was determined by Lowry et al. [1]. For construction of the standard protein curve a series of bovine serum albumin (BSA) dilutions was used (the final concentration ranges between 100–1000 μg/mL). The procedure for protein concentration determination is based on the biuretic reaction of cupric ions (Cu^{2+}) with peptide bonds of the protein in the alkaline environment and the reaction of the phosphomolybdenum–phosphoflavolframic reagent (Folin–Ciocalteu reagent) with aromatic amino acids tyrosine and tryptophan, which are constitutive parts of measured proteins. After binding to the peptide bonds, Cu^{2+} ions are reduced in the cuprous ions (Cu^{+}) and the Cu^{+}-protein complex is formed. This complex further reacts with the added Folin–Ciocalteu reagent to form a blue colored complex. The color intensity is proportional to the protein content and is measured spectrophotometrically at 500 nm.

The activity of SOD was determined by the adrenaline method [2], which is based on the ability of SOD to reduce spontaneous autoxidation of adrenaline in the adrenochrome in the alkaline environment. Autoxidation of adrenaline depends on the presence of $O_2^{\cdot-}$. SOD present in the sample removes $O_2^{\cdot-}$ and thus inhibits the autoxidation reaction. Reduction in the rate of adrenaline autoxidation is determined spectrophotometrically at a wavelength of 480 nm. The change in the absorbance is due to the pink-colored adrenaline. Solutions: 3×10^{-4} M adrenaline in 0.1 M HCl, Carbonate buffer (0.05 M Na_2CO_3 + 10^{-4} M EDTA) pH 10.2 adjusted with 10% HCl and 8 mM KCN. Experimental procedure: 3 mL of carbonate buffer was poured into the glass cuvette, together with an appropriate volume of the pre-adjusted adrenaline and the amount of sample that induces the adrenaline autooxidation inhibition ranging from 16.66% to 66.66%. To calculate the activity of SOD, the value of the sample absorption changes in the blank test (buffer and adjusted adrenaline) was used. The SOD activity unit is defined as the amount of enzyme which leads to 50% inhibition of adrenaline autoxidation in the linear portion of the change in absorbance per minute. The activity of SOD in the samples was expressed in units per milligram of protein (U/mg protein).

The activity of CAT was determined by the method described by Claiborne [3]. The method is based on monitoring the decomposition rate of H_2O_2 to H_2O and O_2 under the action of CAT. Reduction of the absorbance due to the consumption of H_2O_2 was detected spectrophotometrically at a wavelength of 240 nm. Experimental procedure: The H_2O_2 solution in phosphate buffer is adjusted so that the blank sample absorption at a wavelength of 240 nm is between 0.525 and 0.550. In a quartz cuvette, 1.5 mL of the adjusted solution of H_2O_2 in phosphate buffer was poured, and then the amount of sample which leads to a mean change in the absorbance in the range of 0.03 to 0.06 was added. In the sample starts the process of H_2O_2 decomposition reaction due to the presence of CAT. The reduction of the absorbance was monitored spectrophotometrically at 240 nm every 30 s for 3 min at a temperature of 25 °C. To calculate

the CAT activity, a molar extinction coefficient for H_2O_2 (43.6 M^{-1} cm^{-1}), at a wavelength of 240 nm, was used. CAT activity unit is determined as the number of millimoles of H_2O_2 reduced per minute (mmol H_2O_2/min). The activity of enzymes in the tested samples is expressed in units per milligram of protein (U/mg protein).

GSH-Px activity was measured by the method developed by Tamura et al. [4]. The principle of the method is based on the coupled activity of GSH-Px (catalyzes the oxidation of GSH in GSSG with the reduction of organic hydroperoxides) and GR (enables the reduction of GSSG in GSH with oxidation of NADPH as coenzyme). The organic peroxide tert-butyl hydroperoxide is added to the reaction mixture, with NADPH and GR. The activity of the GSH-Px enzyme was detected by spectrophotometric monitoring of NADPH oxidation into $NADP^+$. Into quartz cuvette were poured: 1.6 mL of H_2O (or less volume depending on the amount of added sample), 0.3 mL of 1 mM GSH, 0.6 mL of 0.2 mM NADPH, 0.1 mL of 1 mM NaN3 (which inactivated CAT), 0.1 mL of 1 mM EDTA, 0.3 mL 0,5 M phosphate buffer (pH 7.0), 0.1 mL of 0.03 M tert-butyl hydroperoxide, adequate amount of sample and 5 μL GR. Decrease of the absorbance was monitored spectrophotometrically at 340 nm every 30 s for 3 min at a temperature of 25 °C. The activity of GSH-Px was determined according to the blank, using a molar extinction coefficient 6.22×10^3 M^{-1} cm^{-1} for NADPH at 340 nm. The GSH-Px enzyme activity unit was defined as the number of oxidized nanomoles of NADPH per minute (nmol NADPH/min), and the activity of GSH-Px in the tested tissues is expressed in units per milligram of protein (U/mg protein).

To determine the activity of GR in the tested samples the method according to Glatzle et al. [5] was used. This method is established on the ability of GR to catalyze the GSSG reduction in to GSH with the oxidation of coenzyme NADPH to $NADP^+$. In the reaction mixture the GSSG and NADPH are added, and GR activity is measured spectrophotometrically by reducing the NADPH concentration. Experimental procedure: In a quartz cuvette 0.6 mL 0.5 M phosphate buffer (pH 7.4), 0.1 mL 2 mM GSSG, 0.1 mL 0.5 mM EDTA, 2 mL H_2O (or a smaller volume depending on the volume of the sample) and 0.1 mL 0.1 mM NADPH were added, and at the end adequate amount of sample. The absorbance was measured at a wavelength of 340 nm every 30 s for 3 min at a temperature of 25 °C. To calculate the GR activity, a molar extinction coefficient for NADPH at 340 nm of 6.22×10^3 $M^{-1}cm^{-1}$ was used. The unit of activity of the enzyme GR is defined as the number of nanomoles oxidized NADPH per minute (nmol NADPH/min). The activity of this enzyme in the tested samples is presented in units per milligram of protein (U/mg protein).

The activity of the phase II biotransformation enzyme GST was measured by the method described by Habig et al. [6]. The principle of the method is based on the ability of the GST to catalyze the reaction of binding 1-chloro-2,4-dinitrobenzene (CDNB) to the sulfhydryl group of cysteine that is a part of the tripeptide GSH, thereby forming a CDNB-GSH conjugate. The rate of absorption due to the formation of CDNB–GSH conjugate is directly comparative to the activity of the GST in the sample. Experimental procedure: 2 mL of H_2O poured into the quartz cuvette (or a smaller volume depending on the amount of sample) were followed by 0.1 mL 25 mM CDNB in 95% ethanol, 0.6 mL of 0.5 M phosphate buffer (pH 6.5), 0.3 mL of 20 mM GSH and adequate amount of sample. The absorbance was monitored spectrophotometrically at a wavelength of 340 nm every 30 s for 3 min at a temperature of 25 °C. The GST activity was determined according to the blank, using the molar extinction coefficient for the CDNB–GSH conjugate at 340 nm of 9.6×10^3 M^{-1} cm^{-1}. The absorbance is detected spectrophotometrically at a wavelength of 340 nm. The unit of activity of the enzyme phase II biotransformation GST is expressed as the number of nanomoles of the CDNB-GSH conjugate formed per minute (nmol CDNB-GSH/min). GST activity is given in units per milligram of protein (U/mg protein).

The method described by Griffith [7] was used to measure the concentration of total GSH in the test samples. This method is based on a cyclic enzymatic process: 5,5'-dithiobis (2-nitrobenzoic acid) (DTNB) oxidizes GSH, whereby GSSG and 2-nitro-5-thiobenzoic acid (TNB) are formed, and then the GR enzyme reduces GSSG in GSH with NADPH coenzyme oxidation. The formation rate of the yellow colored TNB compound is monitored and is proportional to the concentration of total GSH in the sample. Experimental procedure: 0.5 mL tissue sonicates and 0.25 mL of 10% sulfosalicylic acid (for protein precipitation in the sample) were poured in microcentrifuge tubes. After centrifugation for 10 min at 5000 rpm, the obtained supernatant was used to determine the concentration of GSH.0.1 mL 6 mM DTNB, sample, 0.7 mL 0.3 mM NADPH, H_2O to 1 mL of reaction mixture and 5 μL of GR were poured in quartz cuvette. For the standards, instead of the sample, the appropriate volumes of standard solutions with known concentration of GSH. Increase of the absorbance is monitored spectrophotometrically at a wavelength of 412 nm every 30 s for 3 min at a

temperature of 25 °C. The absorbance is determined spectrophotometrically at 412 nm. The concentration of total GSH in the sample is expressed in nanomoles per gram of tissue (nmol of GSH/g tissue).

The concentration of free -SH groups in the tested samples was measured by the method of Ellman [8]. DTNB oxidizes free -SH groups present in the sample, whereby mixed disulfides and yellow colored TNB are formed. Experimental procedure: In the cuvette 0.5 ml of sample was added, 0.5 mL of 0.1 M phosphate buffer (pH 7.3) and 0.2 mL of DTNB. After incubation for 10 minutes at room temperature, the absorbance at a wavelength of 412 nm was read. To calculate the concentration of free SH groups, a molar extinction coefficient of 14150 M^{-1} cm^{-1} was used. The concentration of free -SH groups in the sample is determined according to the blank and proportion of formed concentration of TNB. The absorbance is measured spectrophotometrically at 412 nm wavelength. The concentration of free -SH groups in the sample is expressed in micromoles of -SH group per gram of tissue (μmol SH/g tissue).

Thiobarbituric acid reactive substance (TBARS) concentrations as markers of the lipid peroxidation process (LPO) oxidative damage were estimated according to the method of Rehncrona et al. [9]. The content of TBARS formed spontaneously was measured upon treating the samples with cold thiobarbituric acid reagent (10% trichloroacetic acid, 0.6% thiobarbituric acid) and subsequent heating at 100 °C.

The level of protein carbonylation (PC) was determined according to the 2,4-dinitrophenylhydrazine (DNPH) alkaline method [10]. In this approach NaOH is added to the protein solution after the addition of DNPH, shifting the maximum absorbance wavelength of the derivatized protein from 370 to 450 nm. This reduces the interference of DNPH and allows the direct quantification in the sample solution without the need for the precipitation, washing, and resuspension steps that are carried out in the traditional DNPH method.

References

1. Lowry, O.H.; Rosebrough, N.J.; Farr, A.L.; Randall, R.J. Protein measurement with the Folin phenol reagent. *J. Biol. Chem.* **1951**, *193*, 265–275.
2. Misra, H.P.; Fridovich, I. The role of superoxide anion in the autoxidation of epinephrine and simple assay for superoxide dismutase. *J. Biol. Chem.* **1972,** *247*, 3170–3175.
3. Claiborne, A. Catalase activity. In: *Handbook of Methods for Oxygen Radical Research;* Greenwald, R.A., Ed.; CRC Press Inc.: Boca Raton, Florida, USA, 1984; pp. 283–284
4. Tamura, M.; Oshino, N.; Chance, B. Some characteristics of hydrogen - and alkylhydroperoxides metabolizing systems in cardiac tissue. *J. Biochem.* **1982**, *92*, 1019–1031.
5. Glatzle, D.; Vuilleumier, J.P.; Weber, F.; Decker, K. Glutathione reductase test with whole blood, a convenient procedure for the assessment of the riboflavin status in humans. *Experientia* **1974**, *30*, 665–667.
6. Habig, W.H.; Pabst, M.J.; Jakoby, W.B. Glutathione S-transferases. The first enzymatic step in mercapturic acid formation. *J. Biol. Chem.* **1974**, *249*, 7130–7139.
7. Griffith, O.W. Determination of glutathione and glutathione disulfide using glutathione reductase and 2-vinylpyridine. *Anal. Biochem.* **1980**, *106*, 207–212.
8. Ellman, G.L. Tissue sulfhydryl groups. *Arch. Biochem. Biophys.* **1959**, *82*, 70–77.
9. Rehncrona, S.; Smith, D.S.; Akesson, B.; Westerberg, E.; Siesjö, B.K. Peroxidative changes in brain cortical fatty acids and phospholipids, as characterized during Fe^{2+} and ascorbic acid stimulated lipid peroxidation in vitro. *J. Neurochem.* **1980**, *34*, 1630–1638.
10. Mesquita, C.S.; Oliveira, R.; Bento, F.; Geraldo, D.; Rodrigues, J.V.; Marcos, J.C. Simplified 2,4-dinitrophenylhydrazine spectrophotometric assay for quantification of carbonyls in oxidized proteins. *Anal. Biochem.* **2014**, *458*, 69–71.

Table S1. P-values of between aquarium comparisons for each treatment for body length and oxidative stress parameters (one way ANOVA).

**Variable**	***T. macedonicus***		**Hybrid**	
	Shelter	No shelter	Shelter	No shelter
SVL	0.76	0.96	0.11	0.86
SOD	0.24	0.36	0.48	0.29
CAT	0.32	0.87	0.24	0.49
GSH-Px	0.24	0.68	0.38	0.97
GST	0.95	0.87	0.26	0.85
GR	0.43	0.11	0.97	0.30
GSH	0.55	0.27	0.12	0.46
SH	0.13	0.28	0.12	0.70
LPO	0.59	0.99	0.92	0.66
PC	0.38	0.13	0.50	0.27

Table S2. Standardized canonical discriminant function coefficient for the antioxidative parameters.

	**Root 1**	**Root 2**	**Root 3**
SOD	−0.108	0.104	0.460
CAT	−0.578	−0.097	0.753
GSH-Px	−0.313	−0.547	−0.700
GST	−0.029	0.114	−0.347
GR	0.495	−0.744	0.529
GSH	−0.560	0.300	−0.037
Eigenvalue	3.609	0.309	0.074
% of each root	90	8.1	1.9

Table S3. Number of individuals with injuries of different body parts (gills, tail, limbs).

	**gills**		**Tail**		**limbs**	
T. macedonicus	**13**	**$p < 0.001$**	**11**	**$p < 0.001$**	22	$p = 0.298$
Hybrid	**53**		**49**		26	
No shelter	29	$p = 0.121$	29	$p = 0.432$	**30**	**$p = 0.025$**
Shelter	37		31		**18**	

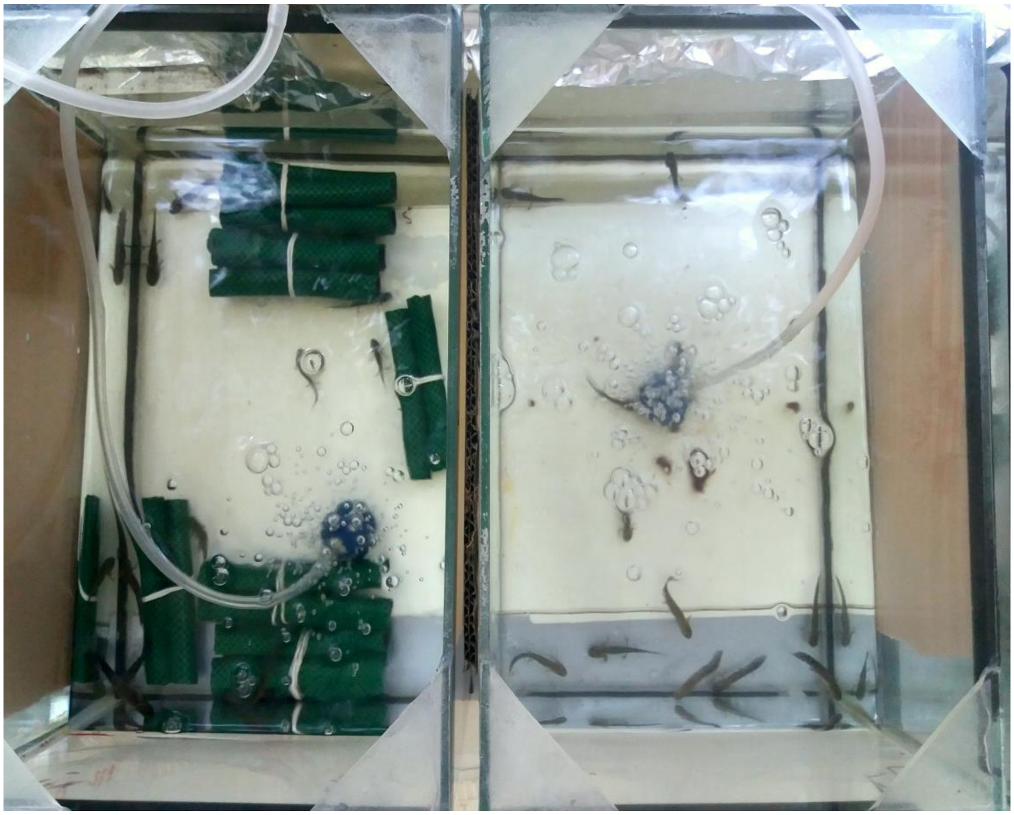

Figure S1. Aquariums with shelter (left) and no shelter (right) conditions.

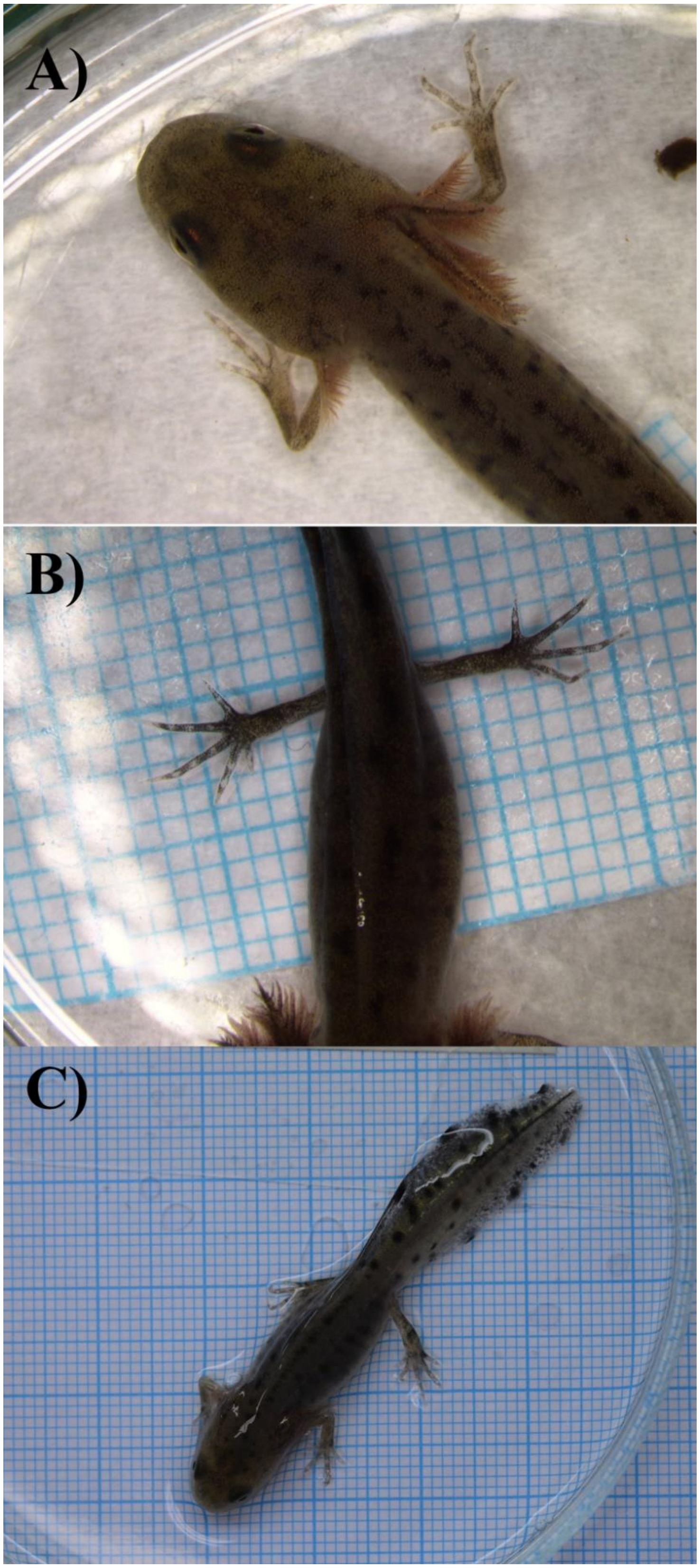

Figure S2. Injuries of gills (A), limbs (B) and tail (C).
